# Supplementary material for: The effect of Drosophila attP40 background on the glomerular organization of Or47b olfactory receptor neurons
Source: G3 (Bethesda). 2023 Jan 25;13(4):jkad022. doi: 10.1093/g3journal/jkad022 (PMC10085800; doi:10.1093/g3journal/jkad022)
Supplement: jkad022_Supplementary_Data [file jkad022_supplementary_data.pdf]

Supplemental Material

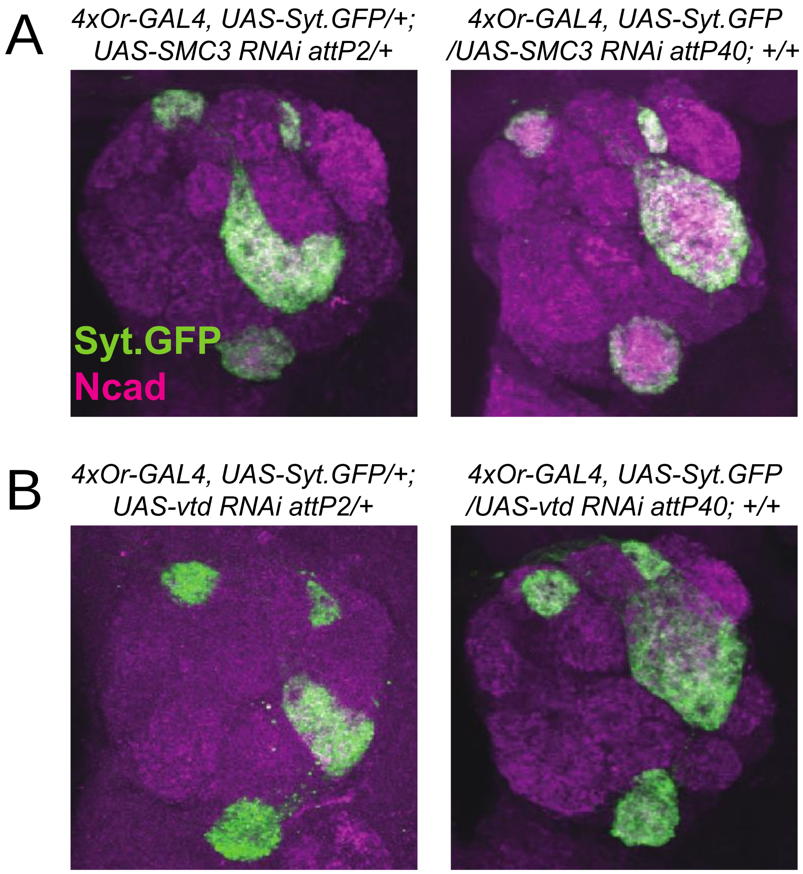

Figure S1. Additional *TRiP UAS-RNAi* results showing the *attP40* but not *attP2*-specific glomerular organization phenotype. Similar to *Beat/Side* screening results in Figure 1A,B, crossing *4xOr-GAL4, UAS-Syt.GFP* chromosome to two *UAS-SMC3 RNAi* lines **(A)** and *UAS-vtd RNAi* lines **(B)** respectively gave rise to glomerular expansion with *attP40* insertion but not *attP2* insertion. 5-9 brains were examined in each genotype and the phenotypical penetrance is 100% in *attP40* groups.

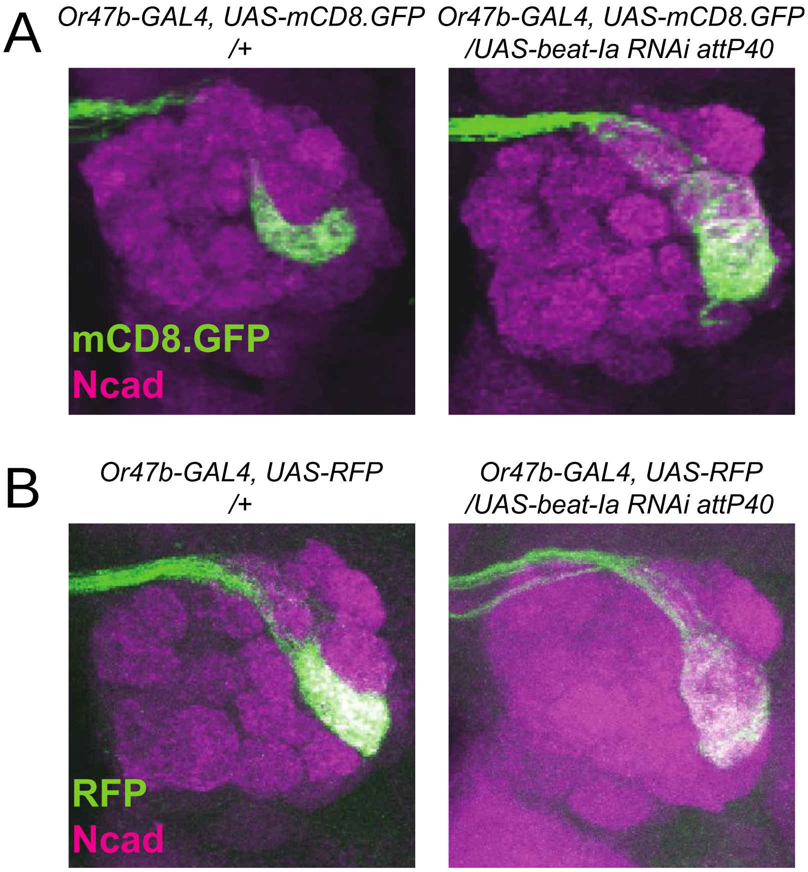

Figure S2. *attP40* over *Or47b-GAL4* recombined with *UAS-reporters* other than *UAS-Syt.GFP* exhibits the same glomerular organization phenotype. *Or47b-GAL4, UAS-mCD8.GFP* **(A)** and *Or47b-GAL4, UAS-RFP* **(B)** over the *attP40* derivative showed VA1v glomerular expansion compared with the respective no-*attP40* control. 5-15 brains were examined in each genotype and the phenotypical penetrance is 100% in *attP40* groups.
